# Wellbeing Convene during COVID-19: A pilot intervention for improving wellbeing and social connectedness for staff, students, residents, and faculty

**DOI:** 10.1017/cts.2023.677

**Published:** 2023-11-08

**Authors:** Farah M. Shroff, Darshan H. Mehta

**Affiliations:** 1 Maternal and Infant Health Canada, Vancouver, BC, Canada; 2 Department of Family Practice, University of British Columbia Faculty of Medicine, Vancouver, BC, Canada; 3 School of Population and Public Health, University of British Columbia Faculty of Medicine, Vancouver, BC, Canada; 4 Harvard HealthLab Accelerators Venture Board Member, Boston, MA, USA; 5 Harvard Medical School, Boston, MA, USA; 6 Benson-Henry Institute for Mind Body Medicine, Massachusetts General Hospital, Boston, MA, USA

**Keywords:** Wellbeing, Faculties of Medicine, burnout, stress management

## Abstract

**Background:**

Canada is facing its worst crisis among healthcare workers in recent healthcare history. Anxiety, depression, suicidal ideation, and severe burnout are higher than before the COVID-19 pandemic. University Faculties of Medicine (FoMs) are vital to healthcare systems. Not only are they responsible for training personnel, but clinicians and staff from FoMs often work directly within healthcare systems. FoMs include students, staff, residents, faculty members, residents, researchers, and others, many experiencing higher stress levels due to pandemic tensions. Most FoMs emphasize cognitive and psychomotor learning needs. On the other hand, affective learning needs are not as well addressed within most FoMs. Finding innovative means to ameliorate mental and emotional health status, particularly at this critical juncture, will improve health and wellness, productivity, and retention. This article discusses a pilot program, *Wellbeing Convene during COVID-19*, in a Canadian FoM, which aimed to (1) provide staff, faculty, residents, and students with a toolkit for greater wellbeing and (2) build a sense of community during isolating times.

**Results:**

Participants found the program beneficial in both regards. We recommend that these kinds of programs be permanently available to all members in FoMs, at no cost. Wellness programs alone, however, will not solve the root causes of mental and emotional stress, often based on concerns related to finances, hierarchical workplace structures, and nature of the work itself, among other factors.

**Conclusion:**

Addressing the mental and emotional health of people in FoMs is vital to improving productivity and reducing stress of FoMs, healthcare professionals, and, ultimately, patients.

## Introduction

The sudden switch from in-person to remote work and financial, health, and other pressures related to the COVID-19 pandemic have created stress within higher-education communities [1]. Faculties of Medicine are diverse–undergraduate students, medical trainees and residents, faculty members, physicians, health care workers, support staff, and more. Long work hours combined with responsibility for people’s lives, a lack of control [2], and a focus on disease lead to stress and even burnout for some clinicians. Additionally, the hierarchical nature of medical institutions and schools has contributed to various negative consequences, such as the inability of those on the lower end of the hierarchy to seek help [3]. Medical students in many countries have experienced extreme stress from mistreatment from senior physicians and faculty members. Toxic dynamics often lead to underreporting and silencing of abusive situations [4]. These hierarchies and stressful workloads result in dynamics that sometimes lead to patient harm [5]. These dynamics were only amplified during the COVID-19 pandemic, as policies typically reinforced existing hierarchies, further rendering residents and others with feelings of powerlessness [6]. The pandemic also exacerbated preexisting tensions within healthcare systems; during the pandemic, demonstrations against healthcare providers have been socially, psychologically, and sometimes physically violent [7–8], promulgating even more burnout and resignations among healthcare workers [9]. This has created the worst crisis in the history of modern Canadian healthcare [10–11].

Furthermore, a complex combination of factors has led to longstanding mental, physical, and emotional health concerns among medical students and physicians, including anxiety, depression, and suicidal ideation [12–13]. These concerns have only heightened during this crisis, leading to higher rates of burnout [14]. Emergency room physicians who identify as women and non-binary, for example, report higher levels of emotional stress than those who identify as males [15]. Wellbeing interventions are needed to address the complex and broad-ranging issues that members of FoMs may face. While most FoMs excel in addressing students’ cognitive, occupational, and social needs, their mental, emotional, and physical needs are often overlooked [16].

This article describes our initiative, *Wellbeing Convene During COVID-19*, a series of webinars for students, staff, faculty, and residents within a Canadian FoM. This eight-month program had the following goals: [1] to improve personal wellbeing and [2] to increase or enhance a sense of community. We were also interested in exploring the role of technology in participants’ mind-body health status during the pandemic.

### Program description: wellbeing convene during COVID-19

The *Wellbeing Convene During COVID-19* Program was a series of mind-body health promotion webinars provided online and for free. These webinars were designed by and for the FoM. We offered 20 webinars that covered a broad range of topics – self-compassion, nutrition, mental health first aid, eye yoga,^1^ enhancing relationships, reflective awareness, dance, leadership and wellness, narrative medicine, and more. The lead author hosted virtually all webinars and presented many of them. Other webinars were presented by colleagues at the same medical school. We received funding from a special initiatives grant within the FoM. Approximately 30 students, faculty, residents, and staff assisted the core team of three people with the planning and delivery of the program. This larger team was surveyed before the curriculum design about activities that would foster wellness in our community. Based on the survey, we emphasized evidence-based practices and showcased local FoM members’ talents. The only exception was a session with an Indigenous elder who was not a member of the FoM.

Programs like *Convene* are part of a global movement in postsecondary institutions, enshrined in the Okanagan Charter, which has an ambitious vision to “transform the health and sustainability of our current and future societies, strengthen communities and contribute to the wellbeing of people, places and the planet.” This initiative encourages the integration of mental, emotional, physical, and other wellness needs into all aspects of campus life [17]. It dovetails with the Edmonton Charter, encouraging higher learning institutions to model a health-promoting and sustainable culture based on social justice principles that support people to make healthy lifestyle choices [18].

Accordingly, postsecondary institutions have provided diverse programing for wellbeing, primarily for students [19–21]. *Convene* was unique in providing programing for students, staff, residents, and faculty together.

## Methodology

### Study design

This study and evaluation are based on the *Wellbeing Convene During COVID-19* program, delivered virtually. We applied a single sample, post-intervention design using multiple measures [22]. Participants were welcome to attend as many of the webinars as they could. At the end of each webinar, we invited them to complete the Qualtrics survey with evaluation questions. Approximately 40–60% of participants completed the evaluation surveys, as they were optional. We anonymously collected post-program questionnaire data from participants to understand the program’s impact on participants’ mind-body health status and subsequent coping mechanisms. We collected short-answer questions, a Likert scale, and yes/no questions. Participants were given time after each webinar to complete the evaluation survey. We were granted ethics approval from the University of British Columbia’s Behavioural Research Ethics Board.

### Recruitment

Participants were faculty members, staff, students, and residents in a FoM. The promotion was carried out through various social media platforms (Facebook, Instagram, Twitter), FoM newsletters, and websites. We also relied on our team of approximately 30 people to circulate webinar information through their channels. Of the approximately 400 people who attended the webinar series, 155 completed evaluation surveys on Qualtrics (response rate = 39%).

### Data analysis

Participants were requested to complete a feedback survey after each webinar asking about their experiences and thoughts. Responses were collected through Qualtrics, and their statistical functions were used to generate a report that provided counts and percentages for our Likert Scale and Yes/No questions. No names were collected so as to maintain anonymity and confidentiality. We used the Qualtrics data selection tools to clean, classify, and merge our data prior to analysis. The text section tool helped us to tag text entry responses with topics for our analysis. All short-answer responses were manually coded for themes and stored on NVIVO, which helped us to manage, control, and find patterns within our data. Themes were derived from an iterative process of mental reflection, analysis, and drawings of mind maps and other diagrams. The themes included connecting with others, outdoor activities, mental wellbeing, spiritual activities, etc.

## Results

### The need for wellbeing and mental health support

While more than 400 people participated in the program, 155 faculty members, staff, students, and residents of the FoM responded to post-webinar surveys. Overwhelmingly, the results indicate that there is a need for more integrative health practices and mental health support and training in this setting:

“[We need more] opportunities to continue connecting with individuals in the FoM!”

“Seminars such as this one go a long way in creating opportunities for being included in activities.”

Some participants also noted that such webinars are an essential component of creating more significant structural changes within the university setting, particularly for research students who work closely with their supervisors:

“Supervisors [bosses] can play a big role in negatively impacting student wellbeing - I think these courses should be targeting supervisors (ideally, it would be mandatory for them to attend).”

“It needs to be normalized that this (wellbeing and looking after ourselves) is important and that output in the form of publications, research, teaching, committee membership, etc. is not the be-all-and-end-all of academia.”

### Overall wellbeing during the pandemic

Since the start of the pandemic, 57% of our participants reported a decline in their overall wellbeing; 34% noted that their wellbeing improved, while 6% felt that their wellbeing had not changed:

“I feel like I’m fine but occasionally will begin to cry.. .”

“I’ve realized that my wellbeing is all connected - my physical, mental, and emotional wellbeing exist together. When I do something to help one of those, it usually helps the others. But there is effort in helping build our own wellbeing, especially in times when we’re struggling.”

Another theme was related to maintaining social networks. Virtually all (99%) participants experienced stress due to isolation, with 10% expressing a great deal of stress and 23% a large extent of stress; 44% feeling moderate, and 22% to a small extent. Narrative responses reflect a range of isolation-induced impacts on physical and mental health:

“Feeling disconnected from people takes its toll.”

### Tools and coping mechanisms during the pandemic

**Figure 1.**
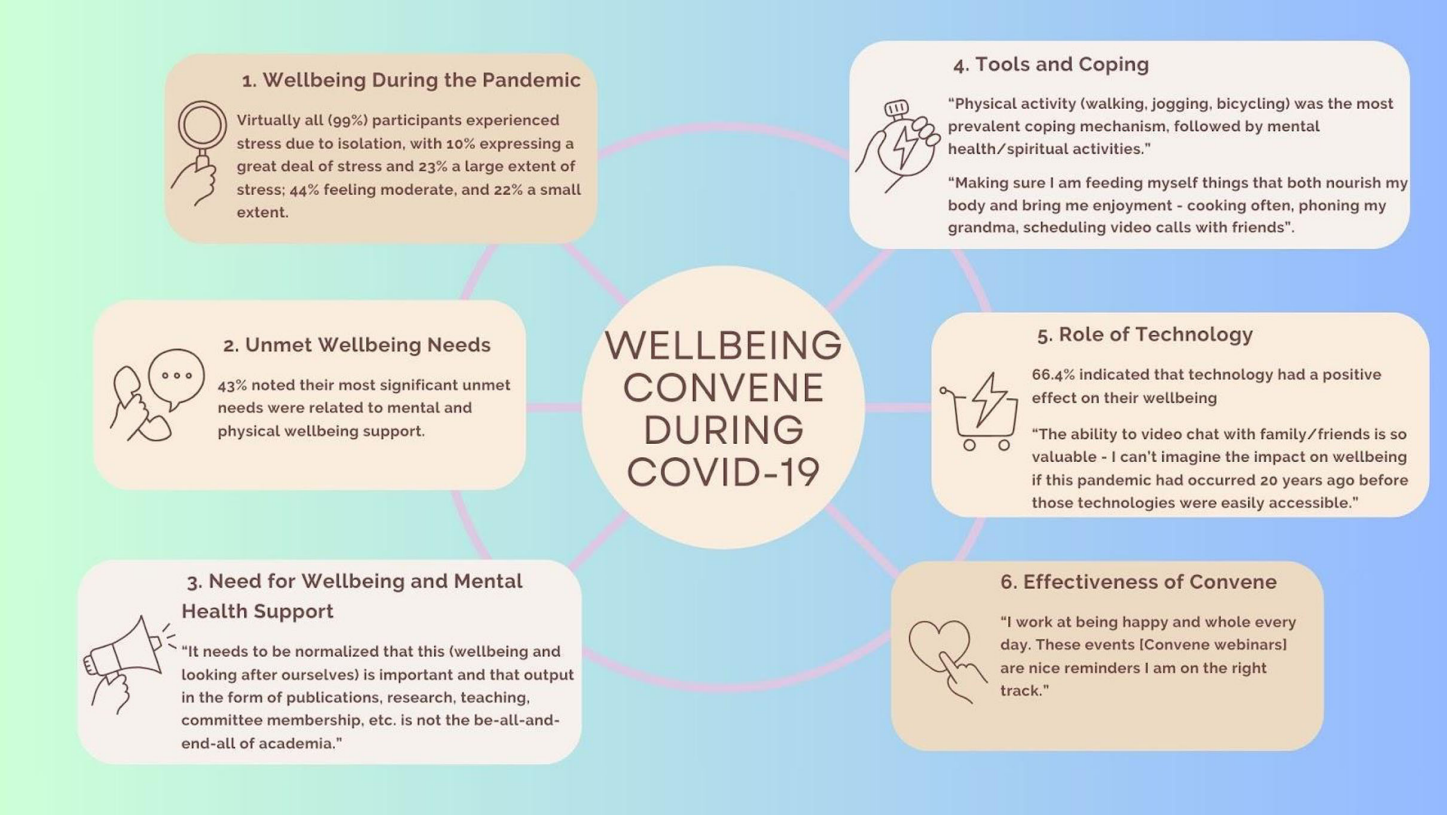
Lessons learned during the implementation and analysis of wellbeing convene (mind map by Brenda Ma, Jaya Kailley, and Simran Grewal).

Most participants reported engaging in multiple activities to maintain or enhance their mind-body fitness. Physical activity (walking, jogging, bicycling, etc.) was the most prevalent coping mechanism. The second most prevalent was engaging in mental health and spiritual activities (meditation, prayer, reflection, etc.), then connecting with others and pursuing personal interests. Figure 1 identifies the most common activities that were used as coping strategies.

Wellbeing activities and coping mechanisms of our participants.

Most participants mentioned multiple activities that contributed to their wellness:

“Making sure I am feeding myself things that both nourish my body and bring me enjoyment - cooking often and trying many new recipes. Phoning my grandma more. Scheduling phone and video calls with friends.”

“Listening to music, listening to podcasts, watching YouTube/ Netflix, ensuring my prescriptions are filled, using reminders on my phone, using digital detox apps, going to bed to ensure a minimum of 8.5 hours of sleep”

### The role of technology

Our 30 *Convene* members were eager to inquire about technology and wellness. Some of them expressed fatigue related to higher amounts of screen time. We discussed the irony of delivering a wellbeing program virtually when people expressed difficulties with technology. We thus asked participants to explain how technology was related to their mind-body health status. Responses were categorized into positive, neutral, negative, and mixed. Most responses (66.4%) indicated that the role of technology was positive – enabling connections for work and social life; in general, technology was considered a vital lifeline during the lockdown.

“The ability to video chat with family/friends is so valuable - I can’t imagine the impact on wellbeing if this pandemic had occurred 20 years ago before those technologies were easily accessible.”

Some participants, approximately 15%, were neutral in their responses:

“Technology has the same role as in usual life”

On the other hand, 13% of participants expressed mixed sentiments:

“It’s a double-edged sword. It has been great to keep me connected, but it is also completely draining to be online in this way, all day!”

“Tech can be wonderful, and it can be harmful. Sometimes I find that I’ll use tech as a coping mechanism for anxious or stressed feelings, to fill a space of seeing friends or going swimming or to an exercise class. But tech also allows me to connect with my friends and family.”

A minority of respondents (5.3%) expressed negative sentiments about the role of technology in their mind-body health status, articulating feelings of exhaustion, frustration, and burnout from excessive technology use:

“If anything, I’ve become more disliking of technology - because I have no choice but to rely on it for my work, I find I don’t want to use it to connect with friends after work, which is a shame (e.g., if I’ve had work Zoom calls all day, I don’t feel like Zooming with my parents or niece in the evening).”

### Effectiveness of wellbeing convene

Most participants (79%) indicated that these webinars were either extremely helpful or very helpful in providing tools and skills to maintain wellbeing and were able to make connections with community members (80%). *Wellbeing Convene* thus appeared to be an effective way of providing wellness tools and building community.

“I work at being happy and whole every day. These events [*Convene* webinars] are nice reminders I am on the right track.”

### Unmet wellbeing needs

Participants noted that their most significant unmet needs were related to mental and physical wellbeing support (43%), followed by academic, financial, and other such issues (16%), relationship building (12%), and work-life balance during remote work (6%). Approximately 15% of participants needed clarification about their unmet wellbeing needs.

## Discussion

*Wellbeing Convene during COVID-19* aimed to improve the mind-body health status of members of the Faculty of Medicine **and** create a sense of community. Post-webinar evaluations sought to understand participants’ coping mechanisms, their use of technology, and other issues during the pandemic. Most of our participants indicated that their wellbeing had declined throughout the pandemic; however, they communicated that they had utilized various coping mechanisms to address their wellness. Data from our questionnaire suggest that the *Convene* webinars helped improve this FoM community’s wellness and that more such programing would address their ongoing wellbeing issues. Figure 1 captures these lessons learned.

While wellbeing programs are offered on campus, most yield a price tag that is not affordable for some community members. Nevertheless, despite the Okanagan and Edmonton Charters, most university campuses do not offer free-of-charge programing [23], particularly for the whole campus community.

### Effective wellbeing programing in postsecondary settings

We could not find literature that comprehensively examined different populations within faculties of medicine, making our work unique because we blended the wellbeing needs of staff, faculty, residents, and medical and graduate students. A 2021 study found that 52% of Canadian medical student respondents were lonelier, and 48% experienced more depression because of the pandemic [24]. This includes residents and faculty members in universities. Our results thus align with that of other studies in confirming that COVID-19 has negatively impacted the mind-body health status of most members in FoMs. We encourage other FoMs to initiate programing that brings together students, staff, faculty, residents, and others.

Virtually all studies within university settings were based on programs geared toward students [25]. One virtual program focused on medical students’ wellbeing skills and connectivity during the pandemic – with many parallels to *Convene*. Students wished this programming to continue after the pandemic [26]. Similarly, an Oxford University 8-week online student mindfulness program reported a reduction in anxiety symptoms [27], consistent with a meta-analysis [28] a trial in Spain [29] and a similar study in China [30]. These studies found that calming activities based on meditation and yoga techniques substantially reduce anxiety, but have less impact on depression.

Stigma, cost, and availability of services prevent many people from seeking treatment. One university tested a digital psychological wellbeing chatbot with positive results, particularly for students who self-reported severe anxiety symptoms. All students noted decreased stress post-intervention [31]. This kind of intervention is worth examining not only for campus mental wellbeing needs but also for the public, given the dearth of human resources to meet ongoing mental health needs.

Others have noted that Canadian universities’ integrative programing should be holistic, allowing for social interaction, creating inclusive and welcoming spaces, and encouraging participants to be engaged in their learning process [32]. *Wellbeing Convene During COVID-19* integrated these aspects within its curricula. Participants reported that programming effectively built relationships and equipped them with wellbeing strategies and coping mechanisms. Our approach was also holistic, as our series of webinars touched upon various topics such as self compassion, mental health first aid, laughter yoga, and other holistic areas.

### Meeting wellbeing needs during the pandemic for campus communities

Campus communities worldwide coped through the pandemic’s isolation in various ways. Like our respondents, physical activity, being in nature, music, art, and connecting with family and friends helped many people manage [33–34]. Unlike our respondents, however, prayer and religious ritual featured large in some campus communities worldwide [35], as did philosophical framing that placed the situation in greater context – life is part of a greater whole [36]. Many students and other members of campus communities found ways to balance and center themselves during this time of unprecedented change.

### Technology’s role in wellbeing

Most participants reported that technology was vital to their wellbeing during the pandemic. Similarly, other studies found that loneliness was abated, while stress and anxiety were reduced through conversations with family and friends over digital platforms [37–38]. Social networking and connections were facilitated virtually, partially meeting people’s emotional and social needs, particularly during lockdowns. Like our participants, most in campus settings voted to continue at least part-time options to work from home, citing less time commuting as an opportunity for self-care and productive work [39–40].

As mentioned by some of our participants, despite the benefits of technology, screen dependency is growing as time spent on devices is increasing [41]. Some of our participants critiqued technology as a necessary part of their workday life but one that brought screen fatigue and other health problems. While surveillance and other human rights issues were not raised, digital technologies invade privacy and collect sensitive data [42]. Literature on campus wellness indicates that technostress is associated with burnout, strain, poor self-regulation, and lower learning agency; students and medical staff exhibited less persistence and engagement in learning, and worked with higher cortisol and lower levels of CoQ10 enzyme – which are correlated with high levels of technostress [43–44]. Higher cortisol and lower CoQ10 enzyme levels are also related to less engagement in work.

Overall, our findings echo and add to the literature suggesting that technology has been a beneficial tool, albeit with side effects, for members of campus communities as they manage the pandemic.

### Improving the wellbeing of those in hierarchies

One of *Wellbeing Convene’s* contributions was integrating students with others who are usually in power over them, including faculty, staff, and residents. By encouraging collective and shared moments of vulnerability, our program brought people together in a way that rarely occurs on campus.

Structural concerns related to mind-body health in the workplace are key. In medicine, steep hierarchies have led to severe mental health concerns, particularly among lower-status students and residents [45]. Nurses and other staff are also negatively impacted by this hierarchy [46]. Without addressing the inherent unfairness of systems that assign power to certain people and take power away from others, wellness programs miss the critical opportunity to address multidimensional stress causes. Making sweeping organizational changes to create workplaces where every voice is genuinely heard engenders wellbeing powerfully and sustainably. A small program such as this one could not make such long-term changes, but issues related to organizational inequities were explored in some sessions. In this time of vulnerability, we found that almost everybody was open to collectively engaging in virtual integrative health activities. We hope that such momentum continues with institutional support.

Programs to address public shaming and other forms of mistreatment of medical students by faculty members have successfully reduced incidences of mistreatment [47]. Other programs have encouraged nurses to speak up using scenarios, personal reflection, and peer support, significantly increasing speaking-up behaviors [48]. Other efforts within clinical settings have likewise succeeded in significant improvements for all members of the healthcare teams’ psychological safety. They have simultaneously improved the learning environment while inculcating support from senior management to ameliorate institutional culture for all healthcare workers, aiming to improve patient care [49]. Programs like *Convene* address self-care and social connections. Ideally, these kinds of programs operate within institutions that aim to create **organizational** resiliency, led by influential members of the institution aiming to instill a culture of organizational justice [50]. Finally, other programs that engage participants in designing their wellness programs from inception to implementation, like Convene, indicate a greater sense of self-control and improved wellbeing [9, 10, 50].

## Conclusion

Most Faculties of Medicine aspire to train physicians and researchers to improve patient and population health. Maintaining and improving the wellness of those within FoMs is a laudable goal and serves to improve morale, support learning cultures, and increase productivity [51]. During times of crisis, such as the COVID-19 pandemic, many opportunities arose. *Wellbeing Convene* took this opportunity to bring many people within one FoM to build wellbeing skills and community. Community members expressed a desire for such programing to remain freely available. The vast majority of wellness programing within postsecondary settings is focused exclusively on students. *Wellbeing Convene*’s innovation was to bring together students, faculty, staff, and residents. We have not found another such inclusive program.

The need for wellbeing programs is very high, as people struggle with serious mental health concerns, workplace stressors, and financial strain related to food, housing, and other costs. Programs like ours cannot address the multifaceted nature of people’s wellbeing needs which also include assessing people’s wellbeing, reducing administrative tasks that create stress, offering work-life balance, leadership training, mentorship, and peer support [52]. However, it is critical to acknowledge the importance of addressing the root causes of stress. *Wellbeing Convene* pointed to the need to address inequities borne out of status differences, but could not comprehensively impact these deeper issues. Programs such as ours, in combination with institutional efforts to create just cultures, would make promising advances in wellbeing. Increasing salaries and benefits would assist in addressing financial needs, as would housing subsidies in places where rents are very high. Addressing issues related to the pressure of grading such as creating more pass/fail courses may be one way of preventing early burnout; providing affordable counseling to students, staff, faculty and others is another solution. Creating large-scale cultural and institutional shifts to root out the causes of moral distress and moral injury, and applying AI to decrease cognitive overload and simplify work functions, are steps towards healthier workplaces [53]. Preventing burnout, in all its dimensions, ought to be a very high priority for FoMs in this time of deep crisis for healthcare systems.

## Footnotes

1 Eye yoga is a part of hatha yoga which applies pranayama, asana, resting, and other yogic practices to relax the eyes and relieve eye strain and fatigue. It consists of moving the eyes to follow finger patterns, blinking, and other movements. Palming, which consists of covering the eyes with the palms, is a key part of resting and relaxing the eyes in eye yoga.
